# Limited Availability of General Co-Repressors Uncovered in an Overexpression Context during Wing Venation in *Drosophila melanogaster*

**DOI:** 10.3390/genes11101141

**Published:** 2020-09-28

**Authors:** Anja C. Nagel, Dieter Maier, Janika Scharpf, Manuela Ketelhut, Anette Preiss

**Affiliations:** Department of General Genetics 190g, University of Hohenheim, Garbenstr. 30, 70599 Stuttgart, Germany; dieter.maier@uni-hohenheim.de (D.M.); Janika_Scharpf@uni-hohenheim.de (J.S.); Manuela.Ketelhut@uni-hohenheim.de (M.K.); a.preiss@uni-hohenheim.de (A.P.)

**Keywords:** co-repressor, C-terminal binding protein, *Drosophila*, Groucho, Hairless, Notch signaling, repressor complex, sequestration, Suppressor of Hairless, wing venation

## Abstract

Cell fate is determined by the coordinated activity of different pathways, including the conserved Notch pathway. Activation of Notch results in the transcription of Notch targets that are otherwise silenced by repressor complexes. In *Drosophila*, the repressor complex comprises the transcription factor Suppressor of Hairless (Su(H)) bound to the Notch antagonist Hairless (H) and the general co-repressors Groucho (Gro) and C-terminal binding protein (CtBP). The latter two are shared by different repressors from numerous pathways, raising the possibility that they are rate-limiting. We noted that the overexpression during wing development of *H* mutants *H^dNT^* and *H^LD^* compromised in Su(H)-binding induced ectopic veins. On the basis of the role of H as Notch antagonist, overexpression of Su(H)-binding defective H isoforms should be without consequence, implying different mechanisms but repression of Notch signaling activity. Perhaps excess H protein curbs general co-repressor availability. Supporting this model, nearly normal wings developed upon overexpression of *H* mutant isoforms that bound neither Su(H) nor co-repressor Gro and CtBP. Excessive H protein appeared to sequester general co-repressors, resulting in specific vein defects, indicating their limited availability during wing vein development. In conclusion, interpretation of overexpression phenotypes requires careful consideration of possible dominant negative effects from interception of limiting factors.

## 1. Introduction

During development, cell fate is adjusted by inter-cellular communication mediated by several signaling pathways including the Notch pathway. Notch is highly conserved from fly to human, having been linked to a number of congenital diseases as well as oncogenesis in humans (reviewed in [1,2]). Hence, there is great interest in the understanding of the molecular mechanisms underlying the regulation of Notch signaling activity, which can be well addressed in the fruit fly *Drosophila melanogaster*. Activation of the Notch pathway by ligand–receptor interaction causes the proteolytic release of the Notch intracellular domain (NICD) that acts as a transcriptional co-activator in the nucleus—together with several additional co-factors, an activator complex is assembled on the Notch target genes driving their transcription. Central to the activator complex is CSL (composite of human C-promoter binding factor 1 (CBF1), fly Suppressor of Hairless (Su(H)), and nematode Lin-12 and Glp-1 phenotype (Lag-1)), a DNA-binding transcription factor that guides the activator complex to Notch target gene promoters (reviewed in [1,3,4,5]). In *Drosophila*, CSL is named Suppressor of Hairless (Su(H)). CSL transcription factors act as a molecular switch since in the absence of receptor activation they assemble repressor complexes on Notch target genes to silence their transcription (reviewed in [5,6,7]). To this end, *Drosophila* Su(H) binds the Notch antagonist Hairless (H), which recruits the general co-repressors Groucho (Gro) and C-terminal binding protein (CtBP), eventually inhibiting Notch target gene expression [8,9,10,11].

Intriguingly, the general co-repressors Gro and CtBP are shared by a number of different repressors that act in various pathways that govern the development of different tissues of *Drosophila* (reviewed in [12,13,14]). Both Gro and CtBP proteins are highly conserved and ubiquitously expressed throughout development to serve their role as general co-repressors [12,13,15,16]. However, because of their joint use by a large number of transcription factors [17,18], it is conceivable that they may be rate-limiting in certain developmental processes or contexts. Within the Notch signaling pathway, these co-repressors serve opposing functions. Firstly, as outlined above, both are assembled in the repressor complex together with Hairless and Su(H) to silence Notch target genes [8,9] (reviewed in [7,10,11]). Secondly, primary Notch target genes belonging to the *Enhancer of split complex* (*E(spl)-C*), encode basic helix-loop-helix proteins that together with Gro act as transcriptional repressors of proneural genes [17,19,20,21,22,23,24] (reviewed in [12,25]). Accordingly, lack of Gro might likewise hamper the repression and the activity of the Notch effectors.

**Figure 1:**
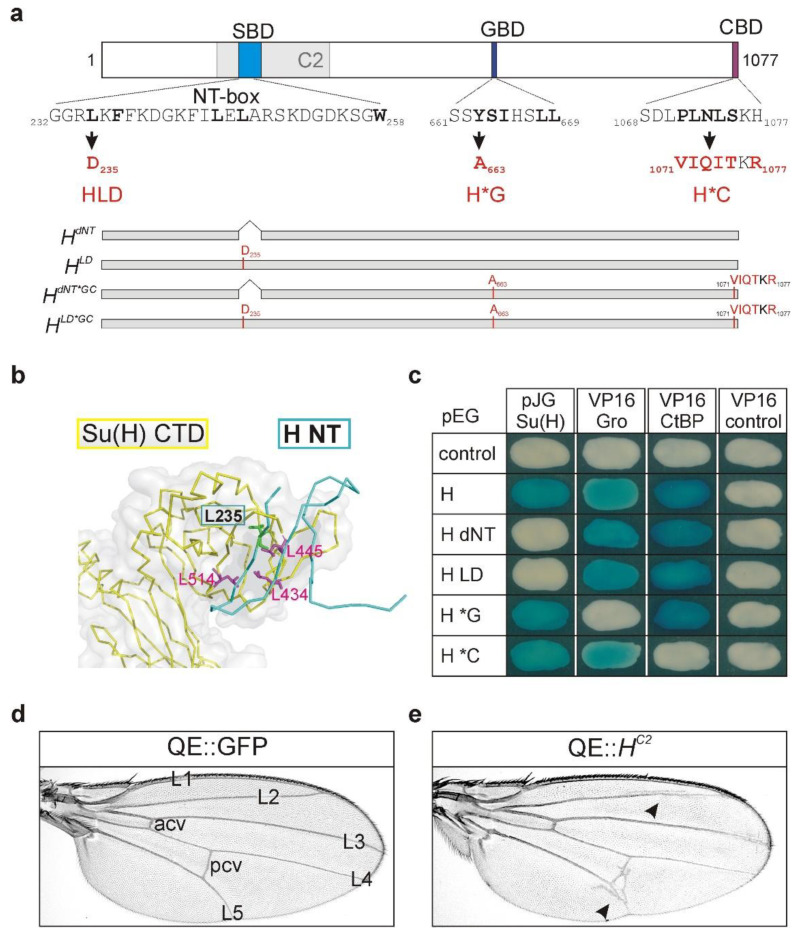
Hairless mutants affecting Su(H) and co-repressor binding. (**a**) Scheme of the H protein with the Su(H)-binding domain (SBD, cyan), the Gro-binding domain (GBD, blue), and the CtBP-binding domain (CBD, purple) indicated. Numbering is according to [55]. The C2 deletion is shown in grey. The NT-box is part of the SBD and contains amino acids in direct contact with Su(H) (bold) [53]. Residues involved in the binding of Gro or CtBP are likewise shown in bold [9]. Amino acid replacements are indicated in red: HLD impairs the interaction with Su(H), H*G prevents binding to Gro, and H*C to CtBP [9,40]. H* mutations used in this study are depicted underneath. (**b**) Structure of the H-Su(H) repressor complex (PDB ID: 5E24) [53]. The picture focuses on the C-terminal domain (CTD) of Su(H) in yellow with amino acids important for H binding shown in pink (L343, L445, L514). The domain in H interacting with Su(H) (H NT, aa 232–269) is shown in cyan; L235 is labelled. (**c**) Pairwise protein–protein interactions assayed by yeast two-hybrid: H wild type or H* mutant constructs in pEG vector served as bait, whereas Su(H) in pJG vector, and either Gro or CtBP in VP16 vector served as prey. Blue colonies reveal pairwise protein interactions, whereas white colonies show respective lack of interaction. Empty vectors served as controls. (**d**) Control wing of a female fly: longitudinal veins L1-L5 and anterior and posterior cross veins (acv, pcv) are labelled (QE-Gal4::UAS-GFP). (**e**) Wing of a female fly overexpressing *H^C2^* in the wing blade (QE-Gal4::UAS-H-C2). Note plexus of ectopic veins along the L2 and L5 veins (arrowheads).

Notch activity is severely downregulated by the overexpression of H because of the repression of Notch target genes. Performed in a tissue-specific manner, H overexpression affects tissue growth, as well as the development of the peripheral nervous system, the eye, and the wing. The resultant phenotypes are all typical for a loss of Notch activity. For example, Notch activity is required for distinguishing dorsal from ventral compartment in the developing wing, and eventually for the formation of the wing margin [26,27]. Compromising Notch activity in this process causes wings to develop characteristic notches, giving the name for the mutant in *Drosophila* [9,26,27,28,29,30]. Moreover, Notch is involved in wing vein formation, restricting vein competent cells to the vein proper [31,32] (reviewed in [33,34,35]). Accordingly, a gain of Notch activity results in gaps, whereas a loss of Notch results in thickened, knotted veins [28,29,31,32,36]. Previously, we noted that the overexpression of a *H^C2^* mutant lacking the Su(H) binding domain induced ectopic veinlets when overexpressed during larval development [36] (Figure 1a,d,e). Formally, this phenotype is unrelated to repression of Notch activity since repressor complex formation was abolished. A systematic genetic analysis, however, revealed a strong interaction with Notch on the one hand, and with the Epidermal growth factor receptor (EGFR) signaling pathway on the other hand [36]. EGFR signaling is tightly involved in vein development of *Drosophila* [31,34,35,36,37]. In general, ectopic veins form when the EGFR pathway is hyperactive, and not are confined to the presumptive veins [30,31]. Interestingly, Groucho has been placed at the crossroads of the Notch and EGFR signaling pathways [38]. Hence, we wondered whether the overexpression of H may sequester the general co-repressors Gro and CtBP, thereby causing the observed phenotypes.

Here, we provide evidence that this is indeed the case in the context of wing vein development. We analyzed mutations in *H* that specifically affect Su(H)-binding (*H^dNT^, H^LD^*), i.e., repressor complex assembly. Accordingly, the overexpression of these constructs does not induce typical Notch loss of function phenotypes expected from excess of wild type *H* gene activity [9,39,40,41]. However, ectopic veinlets formed independent of Su(H) binding activity. We directly tested the hypothesis that venation defects may result from a sequestration of the co-repressors by excessive H protein in this developmental context. To this end, we introduced, in addition, mutations in *H*-destroying co-repressor binding as well. As predicted from the model, overexpression of these *H* mutants defective for binding Su(H), Gro, and CtBP resulted in wings with nearly wild type phenotypes. However, subtle vein defects suggest that H protein may recruit additional co-repressors that are likewise curbed by *H* overexpression. Overall, our data demonstrate that the co-repressors Gro and CtBP are rate-limiting in the context of *Drosophila* wing development.

## 2. Materials and Methods 

### 2.1. Generation of H Mutant Constructs and UAS-Fly Lines Deficient for Su(H) and Co-Repressor Binding

Generation of UAS (upstream activating sequence) constructs containing the wild type H cDNA or the *H^C2^, H^dNT^, H^LD^*, and H^*CG^ mutant versions has been described earlier [9,29,36,40]. Whereas the total Su(H) binding domain is deleted in *H^C2^* and essential parts thereof in *H^dNT^*, the L235D amino acid replacement in *H^LD^* compromises Su(H) binding [40,41,42]. The H^*GC^ mutant carries several amino acid replacements that prevent the binding of both co-repressors, Gro and CtBP (Figure 1a) [9]. These mutations were introduced into already existing UAS-attB *H^dNT^* and *H^LD^* constructs, respectively, by replacement of the C-terminal *EcoR* I DNA fragment with the respective mutant DNA derived from H^*GC^ in order to generate the triple mutant UAS constructs H^dNT*GC^ and H^LD*GC^. Constructs were sequence verified before integration at 68E by PhiC31 mediated recombination, as were earlier the starting constructs H^dNT*GC^ and H^LD*GC^ [40,43]. PCR and diagnostic restriction digests were used for genotyping of the fly strains. Moreover, expression of H protein was confirmed (see Figure S1). The UAS-H^dNT*GC^ and UAS-H^LD*GC^ lines were recombined with UAS-*Su(H)* at 96E by genetic recombination, as outlined before for *H^dNT^* and *H^LD^* [40].

### 2.2. Yeast Two-Hybrid Assays

The procedure for the *Brent* yeast two-hybrid assay has been earlier outlined in detail [44,45]. The pEG202 vector encodes the LexA DNA binding domain to be fused to the bait construct. The pJG4-5 vector encodes the B42 transactivation domain and the VP16-vector of VP16, each to be fused to the prey construct [44,45]. Binding of the two fusion proteins reconstitutes the trans-activator in the transfected yeast cell, resulting in transcriptional activation of the *lacZ* reporter detectable by blue coloration of the yeast on respective X-Gal plates. For the prey constructs, we used pJG-Su(H), VP16-Gro, and VP16-CtBP [9]. The full-length H pEG-bait constructs pEG-H, pEG-H*G, and pEG-H*C were described earlier [9,29]; the full length pEG-HdNT and pEG-HLD constructs were generated in this work. They are based on the respective mutant UAS-H* constructs: the mutant DNA was amplified with Q5 high fidelity DNA polymerase (New England Biolabs) using the primer pair pEG_*H*up 5’ caa tga atc cat ggg ccc tgc t 3’ and pEG_*H*lo 5’ agt gcg aaa atg ttc tta agt cga ctg g 3’, digested at the *Nco* I and *Sal* I sites contained within the primers (bold), and ligated into *Nco* I/*Xho* I-opened pEG 202 vector. All constructs were sequence verified. Empty vectors served as control.

### 2.3. Phenotypic Analyses

The Gal4/UAS system was employed for tissue-specific overexpression [46]. We used the following UAS-H* lines, all placed at chromosomal position 68E on 3L: UAS-*H,* UAS-*H^LD^* [9], UAS-*H^dNT^* [39], UAS-H^dNT*GC^, and UAS-H^LD*GC^ (this work), as well as the respective stocks recombined with UAS-*Su(H)* at 96E [40]. UAS-*H^C2^* [36], UAS-H^*GC^ [47], and UAS-GFP served as control. As driver lines, we used *omb*-Gal4 expressed within the central part of the wing field [48] and QE-Gal4 [49], where Gal4 is expressed in the pattern of the *vestigial* quadrant enhancer, i.e., in the presumptive wing blade only, sparing dorso-ventral and antero-posterior boundaries [50]. Crosses were kept on standard fly food at 25 °C, 20 °C, or 18 °C, as indicated, taking advantage of the temperature sensitivity of the Gal4/UAS system. Variance of phenotypes was rather low (see Figures S2 and S3). Wings of female flies only were dehydrated in ethanol and mounted in Euparal (Roth GmbH, Karlsruhe, Germany). Pictures were taken with either a Pixera 120^es^ or Pro300D camera (Pixera Cooperation, CA, USA) mounted on a Zeiss Axiophot (Carl Zeiss AG, Jena, Germany) using Pixera Viewfinder Pro2.5 software. Pictures were assembled using Photo Paint and Corel Draw software.

## 3. Results

### 3.1. H Constructs Deficient for Su(H) and Co-Repressor Binding 

The H protein is characterized by three interactive motifs: the Su(H)-binding domain (SBD) contacts Su(H) with its N-terminal (NT)-box, whereas the Groucho-binding domain (GBD) and the CtBP-binding domain (CBD) contact the co-repressors Gro and CtBP, respectively (Figure 1a) [8,9,39,40,51,52]. Recently, the crystal structure of the H-Su(H) repressor complex uncovered an unusual hydrophobic interaction between the two proteins—H NT deeply reaches into the C-terminal domain of Su(H), where primarily leucine residues are in contact with each other (Figure 1b) [53]. To address the contribution of either component to *H* function, mutations have been introduced into the respective binding sites (Figure 1a,b) [9,40,42]. The H*G and H*C point mutations indicated in Figure 1a affect the contact sites for Gro and CtBP, respectively [12]. We have shown earlier that these mutations impede co-repressor recruitment by H, and accordingly the strength of H-mediated repression [9,47,54]. Indeed, in the yeast two-hybrid assay, H*G does not bind to Gro, and H*C does not bind to CtBP, whereas the binding to Su(H) was unaffected (Figure 1c) [9].

Accordingly, mutations within the H-Su(H) interface affected the interaction between the two proteins in a yeast two-hybrid assay (Figure 1a–c). For example, HdNT lacks the NT-box (amino acids G232 to S270) including most relevant amino acids required for the binding of Su(H) [39,40,53]. In HLD, the amino acid leucin_235_ within the Su(H) interface was replaced by the bulky and charged aspartate residue, expected to interfere with Su(H) binding (Figure 1a,b) [40,42,53]. Correspondingly, HLD did not bind to Su(H) in the yeast two-hybrid assay (Figure 1c). Interaction with either co-repressor Gro or CtBP, however, was not influenced by any of the mutations impairing the Su(H) interface (Figure 1c).

### 3.2. Overexpression of Su(H) Binding-Deficient H Proteins Resulted in Ectopic Veinlet Formation

Earlier, we showed that the overexpression of a *H^C2^* variant induced a specific wing phenotype characterized by a plexus of ectopic veinlets [36], which we also observed when overexpressing *H^C2^* in the presumptive wing field proper (Figure 1d,e). To this end, we employed the Gal4/UAS system for tissue-specific overexpression of *H^C2^* using the QE-Gal4 line [46,49]. In this line, the *vestigial* quadrant enhancer controls expression within the entire wing field, sparing antero-posterior and dorso-ventral boundaries [50]. The H^C2^ mutant protein is unable to bind to Su(H), i.e., to assemble repressor complexes [9]. Accordingly, downregulation of Notch activity can hardly explain the *H^C2^* overexpression phenotypes. Rather, genetic interactions point to an interference with EGFR signaling activity [36], perhaps by interfering with co-repressor availability. The substantial size of the *H^C2^* deletion (Figure 1a) moreover raised the possibility that *H^C2^* may have defects in addition to Su(H) binding relevant to the observed phenotypes. We hence wondered whether H* variants affecting Su(H) binding in particular might elicit similar phenotypes. On the basis of the structure analysis, respective mutations have been generated previously, allowing us to directly address this question (Figure 1a–c) [40,42,53].

First, we aimed to address the biological activity of the Su(H)-binding deficient H* mutants UAS-*H^dNT^* and UAS-*H^LD^* with regard to vein development. We and others have shown before that the overexpression of *H* induces strong developmental defects that result from a repression of Notch activity [9,26,29,39,40,41,47,49,52,56,57]. Phenotypic strength can hence serve as readout of residual H activity in an overexpression context. To avoid position effects and allow for a direct comparison of the respective constructs, we placed UAS-*H^dNT^* and UAS-*H^LD^* at the identical chromosomal position on the left arm of the third chromosome at position 68E by PhiC31-mediated integration, as outlined previously [40,43]. Expression of the constructs was confirmed (see Figure S1).

**Figure 2:**
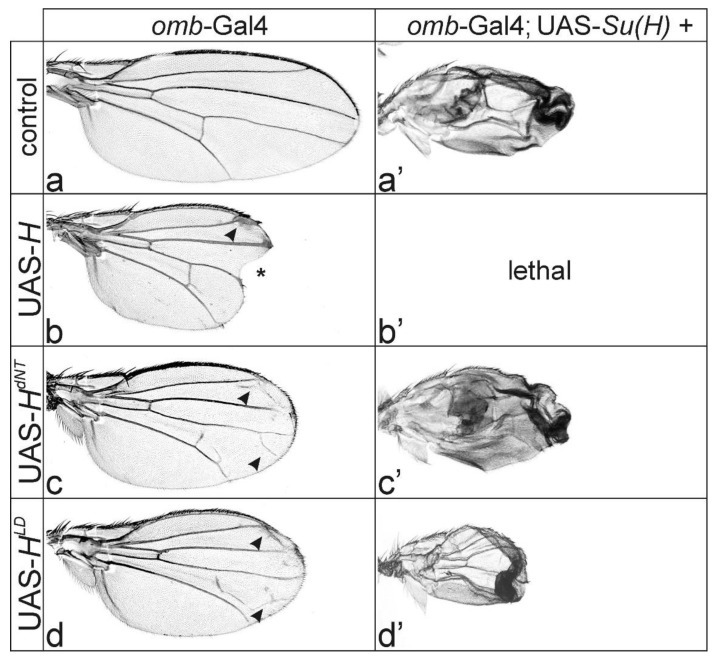
Overexpression of H protein variants in the central wing anlagen using *omb*-Gal4. (**a**–**d’**) The transgenes, as indicated, were overexpressed in the central part of the wing anlagen using *omb*-Gal4 at 20 °C. Female wings are shown. (**a**) UAS-GFP served as control. (**b**) Wing incisions (asterisk), proliferation defects, and vein thickening (arrowhead) were typical consequences of an inhibition of Notch activity resulting from overexpression of wild type H protein. (**c**,**d**) Overexpression of either *H^dNT^* or *H^LD^* mutants did not affect the wing margin. Note, however, ectopic wing vein formation (arrowheads mark examples) and slightly reduced wing size. (**a’**–**d’**) Combined overexpression of *Su(H)* and *H* variants. (**a’**) Whereas the overexpression *Su(H)* within the wing anlagen resulted in crumbled wings, a combination with wild type *H* was lethal as a consequence of excessive repressor complex formation (**b’**) [9,40,52,53]. In combination with either (**c’**) *H^dNT^* or (**d’**) *H^LD^*, however, the wings resembled those of the sole *Su(H)* overexpression.

The principal H activity is the assembly of repressor complexes at Notch target gene promoters. Accordingly, the overexpression of H* transgenes deficient for the binding to Su(H) has little developmental consequences. As shown earlier, the selection of mechano-sensory organs, growth as well as differentiation of the eye were largely normal in an overexpression context of either *H^dNT^* or *H^LD^* mutants [40,41]. Likewise, the formation of the wing margin, which requires a specific activation of Notch and its targets *wingless* and *vestigial* along the dorso-ventral boundary in the wing imaginal disc [26,50], was not affected by the overexpression of either *H^dNT^* or *H^LD^* mutant [40]. Whereas the overexpression of wild type *H* in the central wing anlagen with *omb*-Gal4 induced strong wing incisions (Figure 2a,b) [9,40], the wing margin developed normally, inducing either *H^dNT^* or *H^LD^* in excess (Figure 2a–d). Instead, a subtle wing vein phenotype was observed—ectopic veinlets appeared in the distal part of the wing in the intervein regions reminiscent of what has been seen with *H^C2^* (Figure 1e and Figure 2c,d).

### 3.3. Ectopic Vein Formation Was Independent of Su(H) 

**Figure 3:**
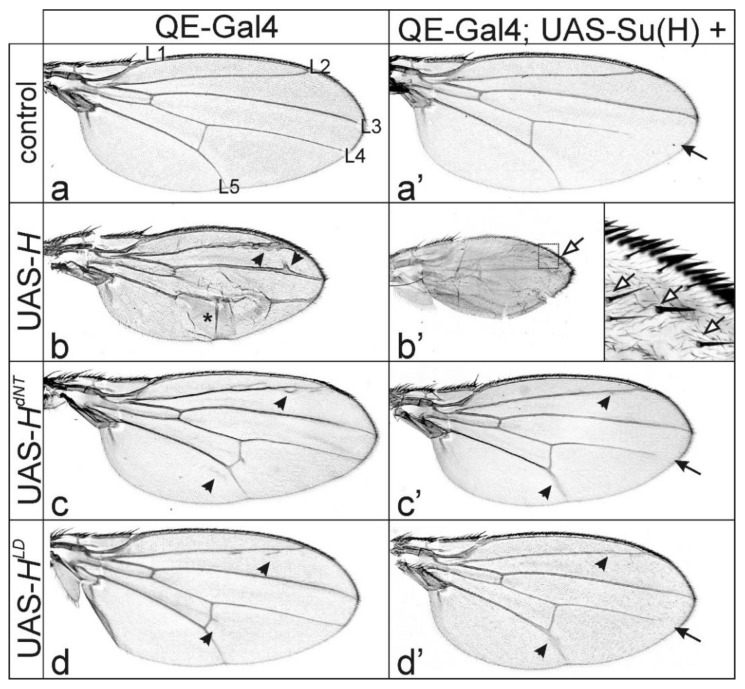
Overexpression of H protein variants in the presumptive wing tissue using QE-Gal4. (**a**–**d’**) UAS-H* transgenes, as indicated, were overexpressed in the presumptive wing tissue using QE-Gal4 at 18 °C. (**a**) UAS-GFP served as control; longitudinal veins are numbered. (**b**) Overexpression of wild type H protein affected wing size and tissue adhesion (asterisk). The most prominent phenotype, however, was a thickening of veins and formation of ectopic veinlets (arrowheads point to examples). (**c**,**d**) The latter was also observed upon the overexpression of *H^dNT^* (c) or *H^LD^* (d) variants compromised in Su(H) binding (arrowheads point to examples). (**a’**–**d’**) UAS-*Su(H)* plus the respective UAS-H* transgenes were overexpressed in combination in the developing wing field with QE-Gal4. (**a’**) *Su(H)* overexpression alone caused a loss of distal parts of longitudinal veins (arrow). (**b’**) A combined overexpression of UAS-*Su(H)* with UAS-*H* resulted in a transformation of intervein to vein tissue, as well as the emergence of ectopic bristle organs (open arrow, see enlargement). Combination of *Su(H)* with either *H^dNT^* (**c’**) or *H^LD^* (**d’**), however, gave a mixed phenotype with erased distal wing veins (arrow) plus ectopic veinlets (arrowheads), indicating lack of repressor complex formation. Female wings are shown.

To further analyze the venation defects, we switched to the QE-Gal4 line for expression within the presumptive wing field [49]. Overexpression of *H* with QE-Gal4 resulted in thickened veins and smaller wings compared to control (Figure 3a,b). In addition, a partial detachment of the dorsal and ventral wing epithelia was frequently observed, causing wing blisters (Figure 3b). In contrast, overexpression of any of the two Su(H)-binding defective H* transgenes *H^dNT^* or *H^LD^* induced knotting of the longitudinal vein L2 and ectopic veinlets at the posterior cross-vein and along the longitudinal L2 and L5 veins (Figure 3c,d). This phenotype is remarkably similar to *H^C2^* overexpressed in the quadrant pattern (Figure 1d,e), in support of the idea that overexpression of H* isoforms defective for Su(H)-binding elicit these effects.

If there were residual binding between the mutant H* proteins and Su(H), the observed phenotypes might still be a consequence of Notch signaling repression. Overexpression of *H* strongly downregulates Notch activity, causing typical Notch loss of function phenotypes such as thickened veins [29,32]. Notably the knotted L2 veins seen in the QE-Gal4-induced wings are reminiscent of the sole *H* overexpression (Figure 3b), in support of residual *H* activity in the mutants. In this case, we would expect a more severe phenotype to arise from a combined overexpression together with *Su(H)*. As observed by several groups before, the co-overexpression of *H* and *Su(H)* results in an extreme repression of Notch activity, and hence strong phenotypes arise, including larval or pupal lethality [9,40,52,53,57]. Indeed, co-overexpression of *H* with *Su(H)* in the central wing anlagen using the *omb*-Gal4 driver line caused pupal lethality even at lower temperature (Figure 2b’). In contrast, combinations with *H^dNT^* or *H^LD^* were viable, and the resultant wings resembled those of the sole *Su(H)* overexpression (Figure 2a’,c’,d’), in support of a failure of repressor complex formation with any of the mutant H* isoforms together with *Su(H).*

Overexpression of *Su(H)* with QE-Gal4 caused shortening of longitudinal veins at their distal ends, easily explained by a gain of Notch activity (Figure 3a’) [32,33,34,35,36]. In combination with *H*, *Su(H)* overexpression caused much smaller wings with extremely broadened L2 and L5 veins, as if the entire provein field had turned into vein character (Figure 3b’), a typical sign of strong Notch inhibition [32]. Moreover, accessory mechano-sensory bristles developed along these veins (enlarged in Figure 3b’), indicating gain of proneural gene activity. In contrast, the combination of *Su(H)* with either *H^dNT^* or *H^LD^* did not affect wing size, only causing a slight knotting of distal L2 and L5, and, occasionally, a gap in distal L4 or L5, i.e., a mixed phenotype (Figure 3c’,d’). Together these data show that the mutant proteins H^dNT^ and H^LD^ have lost Su(H)-binding activity, however, they still elicit the formation of ectopic veinlets. However, we should note that direct in vivo evidence of non-binding is lacking.

### 3.4. Overexpression Phenotypes Were Dependent on Co-Repressor Recruitment 

**Figure 4:**
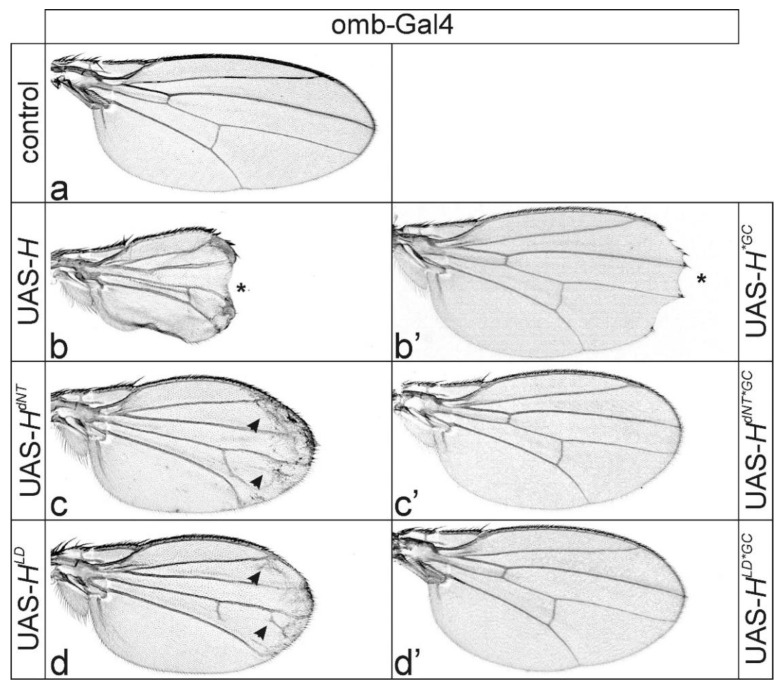
Loss of co-repressor binding inhibited ectopic vein formation by H protein overexpressed in the central wing anlagen. (**a**–**d’**) UAS-H* transgenes, as indicated, were overexpressed in the central part of the wing anlagen using *omb*-Gal4 at 25 °C. Note enhancement of phenotypes at the higher temperature (compare with Figure 2). (**a**) UAS-GFP served as control. (**b**) Wing incisions (asterisk) are very deep when *H* is induced. Overexpression of either *H^dNT^* (**c**) or *H^LD^* (**d**) isoforms, however, induced a lattice of veinlets (arrowheads point to examples). The margin was unaffected, however. (**b’**–**d’**) Overexpression of the respective H* protein isoforms lacking Gro and CtBP co-repressor binding in addition to compromised Su(H) binding (*GC; see Figure 1). (**b’**) Loss of co-repressors impeded H repressor activity. Accordingly, mild wing incisions were seen when UAS-H^*GC^ was overexpressed. In contrast, overexpression of either H^dNT*GC^ (**c**) or H^LD*GC^ (**d**) variants affecting both Su(H) and co-repressor binding neither affected wing margin nor vein development. Female wings are shown.

**Figure 5:**
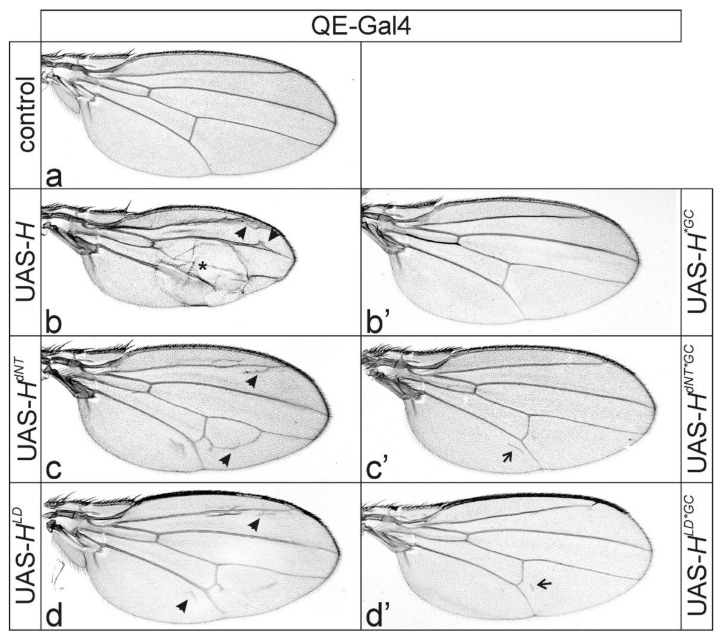
Overexpression of H* protein lacking Su(H) and co-repressor binding ability in the developing wing blade. (**a**–**d’**) UAS-H* transgenes, as indicated, were induced at 25 °C with QE-Gal4 in presumptive wing tissue. (**a**) UAS-GFP served as control. (**b**) Whereas wild type H induced wing blisters (asterisk) and thickened veins (arrowheads), H^dNT*GC^ (**c**) and H^LD*GC^ (**d**) isoforms induced ectopic veinlets (arrowheads point to examples). (**b’**–**d’**) Overexpression of the respective H* protein isoforms that in addition lack Gro and CtBP co-repressor binding (*GC; see Figure 1a). Overall, the wings resembled the control. However, minute veinlets may appear in the developing wing blade. Examples are shown for (**c’**) H^dNT*GC^ and (**d’**) H^LD*GC^ (arrows). Female wings are shown.

We wondered whether excessively overexpressed H* protein might intercept the activity of its co-repressors Gro and CtBP. Both co-repressors are recruited by a multitude of other repressors [12,13,17,18], and if sequestered, may affect vein formation. In this case, H proteins lacking both co-repressor binding as well as Su(H) binding should not induce wing venation phenotypes. To this end, the respective triple mutant UAS lines were established (H^dNT*GC^ and H^LD*GC^) (Figure 1a), as well as the constructs overexpressed in the central part of the wing anlagen using *omb*-Gal4 (Figure 4) and in the presumptive wing field using QE-Gal4 (Figure 5).

We have shown before that H^*GC^ strongly affects H repressor activity during wing and eye development [9,47,54], in agreement with the pivotal role of the co-repressors in repressor complex functions. Accordingly, the overexpression of UAS-H^*GC^ with *omb*-Gal4 affected wing margin formation to a much lesser degree compared to UAS-*H* (Figure 4b,b’). Moreover, overexpression of H^*GC^ with QE-Gal4 allowed normal wing venation (Figure 5b,b’). Strikingly, nearly normal wing vein development was observed upon the overexpression of any H* mutant affecting both *Su(H)* and co-repressor binding—neither extensive ectopic vein formation nor vein knotting was observed (Figure 4c–d’ and Figure 5c–d’). Wing size, however, albeit significantly improved compared to Su(H)-binding defective H* mutants, was still markedly reduced relative to control wings when overexpressing the triple mutants H^dNT*GC^ and H^LD*GC^ in the *omb* pattern (Figure S2). Moreover, we still noted subtle venation defects when inducing either H^dNT*GC^ or H^LD*GC^ with the QE-Gal4 at higher temperatures in about half of the specimens (Figure 5c’,d’ and Figure S3). These phenotypes may point to additional co-repressors likewise involved in wing vein development. When contacting H at sites other than Gro and CtBP, such co-repressors are expected to bind to the mutant H* isoforms and might be likewise depleted by H* overexpression. Whether these include known chromatin remodeling factors such as Asf1 or still others remains to be determined [58,59].

## 4. Discussion

Formation of wing vein primordia and differentiation of veins are controlled primarily by three signaling pathways in *Drosophila*, namely, EGFR, Notch, and Decapentaplegic (Dpp, i.e., Transforming growth factor ß-like) [33,34,35,60]. No veins develop in the absence of either EGFR or Dpp, or in excess of Notch activity [33]. During larval development, EGFR activity defines pro-vein and inter-vein territories, and the increase in EGFR activity induces a plexus of ectopic veins [30,33]. Notch activity, however, refines pro-vein territories to the veins proper. Accordingly, downregulation of Notch results in thickened and knotted veins, whereas upregulation inhibits vein formation altogether [34,35]. Activity of Dpp is required in the pupal stage for the initiation and maintenance of the vein differentiation program. In addition, several downstream transcription factors eventually specify the final veins [34,35,60,61,62].

We provide evidence that the overexpression of H* mutant protein incapable of Su(H)-binding causes the formation of ectopic veinlets by sequestration of co-repressors. Notably, Gro has been shown before to be pivotal to the regulation of EGFR-signaling activity in the wing, and has been placed at the crossroads of the Notch and EGFR signaling during embryogenesis as well as wing development of *Drosophila* [36,38,63]. In fact, Gro is a target of Mitogen-activated protein kinase (MAPK) activity downstream of EGFR activation, and phosphorylation by MAPK impedes Gro repressor capacity [38]. In addition, EGFR signaling activity may directly intercept with Notch signaling at the level of H and Su(H) as well, since either protein is a phospho-target of MAPK in *Drosophila* [49,64], as is CSL in the mouse [65].

**Figure 6:**
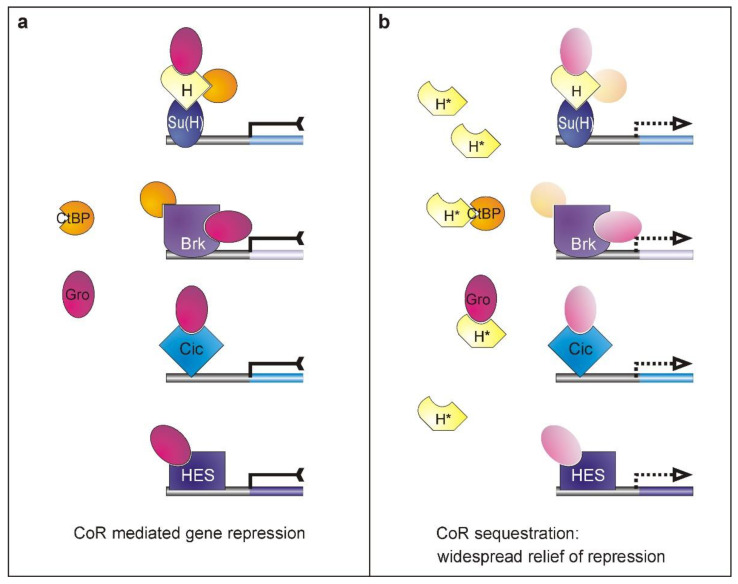
Model explaining the ectopic veinlet phenotype. (**a**) General co-repressors Gro and CtBP are shared by numerous regulators acting in different signaling pathways, including Brinker (Brk), Capicua (Cic), and E(spl) and Hairy (HES)-type transcription factors. Moreover, Hairless recruits both co-repressors for Su(H)-H repressor complex formation. (**b**) Overexpression of H* variants defective for Su(H)-binding does not influence Notch target gene expression any longer. H* variants, however, may compete with other transcription factors for the binding to co-repressors, thereby limiting their availability. As a consequence, H* overexpression may intercept with signaling pathways involved in vein formation that are specifically sensitive towards a mitigation of co-repressor availability.

We propose a model, whereby excessive H* protein defective in Su(H)-binding—and hence unable to form repressor complexes to silence Notch target genes—sequesters general co-repressors Gro and CtBP (Figure 6). Ectopic veinlets form because of limited availability of the co-repressors rather than because of a specific H activity. Both co-repressors, Gro and CtBP, are shared by numerous transcription factors acting in different pathways, including Notch, EGFR, and Dpp pathways (reviewed in [12,13,25]). Presumably, the widespread relief of repression underlies the observed phenotypes, rather than the interception of a single, specific transcription repressor. This hypothesis is in line with the observation that a vast majority of transcription factors (around 80%) involved in wing development recruit the co-repressors Gro and/or CtBP [66]. These data were deduced from a large-scale in vivo screen, where transcription factors were either overexpressed during wing development or downregulated by RNA interference, and wing phenotypes recorded [66]. Comparing the author’s results with the list of physical interaction partners curated in *flybase*, we found that 85% of Gro partners and 75% of CtBP partners induced wing defects. Accordingly, limited availability of Gro and/or CtBP may affect the activity of many different transcription factors at once. We consider Capicua (Cic), Brinker (Brk), and E(spl)/Hairy the most likely candidates, acting in EGFR, Dpp, and Notch signaling pathways, respectively, and each involved in wing vein development [30,36,62,66,67] (Figure 6).

### 4.1. Influences on EGFR Signaling Activity 

It is well established that the upregulation of EGFR signaling activity results in ectopic vein formation [30,31,33,68]. Either gain of EGFR activity or the loss of one of its antagonists may induce ectopic veinlets [67,68,69,70,71]. For example, *capicua (cic)* encodes a transcriptional repressor downstream of EGFR and of Dpp signaling activity. Cic is required for the specification of intervein fate by acting as a repressor—together with its co-repressor Gro—of vein-specific genes, for example *vvl, aos,* and *dpp* [67,72]. In the absence of Gro, Cic loses its silencing activity. Accordingly, *cic* mutants display numerous ectopic veins specifically along the L2 and the distal L4 and L5 veins [67], i.e., exactly in the regions where we observed ectopic veinlets (Figure 1e). Moreover, cell clones mutant for *Gro* display extra veins, similarly to *cic* clones [63]. Cic repressor function may be affected in cases of restricted Gro availability, and ectopic veins would result (Figure 6).

### 4.2. Influences on Dpp Signaling Activity 

During pupal development, *dpp* signaling activity is required in cells committed for vein fate. Expression of *dpp* is prefigured by EGFR and Notch activities, defining the presumptive vein territories. In fact, *dpp* activity is sufficient for inducing vein differentiation within the wing field [33,62]. As outlined above, a Cic–Gro repressor complex inhibits expression of *dpp*, and hence Dpp signaling activity [67]. Moreover, the activity of *dpp* is confined by Brinker (Brk) that is specifically expressed in the interveins [62]. Brk acts as a transcriptional repressor and recruits both co-repressors Gro and CtBP [73,74]. Brk-induced repression of Dpp during wing development can elicit venation phenotypes that are very similar to H* overexpression [75,76]. Assuming limited availability of either or both co-repressors, Brk’s activity might be impeded, lifting Dpp repression, and hence allowing for the formation of ectopic veinlets (Figure 6).

Similarly, the repressor Scribbler (Sbb; also named Master of thickveins, Mtv) and its co-repressor Gro influence Dpp activity through the regulation of the Dpp receptor Thickveins. Accordingly, cells lacking *sbb* execute vein fate [77], which might also be expected in the absence of Gro [63]. However, *sbb* was not amongst the transcription factors recovered in a systematic in vivo screen for genes regulating wing development [66]. Another transcription repressor downstream of Dpp is Knirps (Kni). Kni is specific to the formation of the L2 vein, recruiting both co-repressors Gro and CtBP [78,79]. Absence of co-repressors is expected to result in gaps rather than ectopic veins.

### 4.3. Does H Recruit Additional Co-Repressors?

Our data provide evidence that H may recruit additional co-repressors apart from Gro and CtBP, since the overexpression of the triple mutants H^dNT*GC^ and H^LD*GC^ still affected wing size (Figure 4) and induced some remnant veins (Figure 5). Residual binding to Su(H) appears an extremely unlikely explanation, as H^dNT*GC^ deletes most of the Su(H)-binding domain (Figure 1a), and co-expression of *Su(H)* did not enhance the observed phenotypes (Figure 2c’,d’). Formally, H* may compete with the primary Notch signaling executers, the HES protein family (Hairy-Enhancer of split) for binding to the Gro co-repressor. In the Notch signaling pathway, Gro serves as co-repressor in opposing ways: firstly, together with H in Notch target gene repression, and secondly, as co-repressor of the bHLH proteins encoded by the primary Notch targets of the Enhancer of Split E(spl)/HES family (reviewed in [10,25,80]). When Notch signals are executed, they are involved in eventually refining vein width [32,33,34,35]. In case of limited Gro availability, their activity might be reduced, and ectopic veins may be a consequence (Figure 6).

Additional co-repressors of H may involve chromatin modifiers such as anti-silencing factor 1 (Asf1), and indirectly Sin3A and Sirtuin 1 (Sirt1) [58,59,81]. Asf1 is a nucleosome remodeler involved in epigenetic regulation of Notch signal transduction, binding to H in this context [58,59]. As part of multi-protein complexes, Asf1 together with histone deacetylases Sin3A and HDAC1 is involved in the silencing of Notch target genes [58,59]. Recently, however, Sin3A was shown to promote rather than repress Notch signaling activity during wing margin formation [82]. Su(H) directly contacts Sin3A as well as the Sirt1, whereas Asf1 binds to Gro, adding additional complexity to possible competition amongst co-repressors [58,59,81]. During wing development, Sirt1 has a positive effect on Notch activation in *Drosophila* and participates in the deacetylation of Su(H) [81]. Sirt1 mutant wings develop ectopic veins similar to H* overexpression mutants [81]. Accordingly, a limitation of Sirt1 availability by H* overexpression is in agreement with the observed residual ectopic veinlets. In addition to a direct effect on Su(H) activity by deacetylation, the role of Sirt1 during wing vein development may be indirect and involve the epigenetic gene regulator Kismet (Kis). Kis encodes an ATP-dependent chromodomain transcription factor that interacts with Sirt1 [81,83]. Kis was originally identified as a genetic modifier of EGFR activity, and loss of *kis* leads to the development of ectopic veins due to elevated levels of EGFR signaling activity [68,84]. Moreover, Sirt1 forms a repressor complex with the lysine-specific demethylase LSD1/Su(var)3-3, regulating Notch and Dpp signaling pathways, respectively, in the developing wing [85,86,87]. Along with Sirt1, CoREST, CtBP, HDAC1/2, and others, LSD1 assembles in a repressor complex mediating histone modifications [88]. Intervein cells mutant for either *lsd1* or its cofactor *CoRest* adopt vein cell fate, indicating that the complex functions to repress vein formation [86,87]. Limited availability of CtBP by intercepting H* protein may likewise impede Sirt1–LSD repressor complex function, followed by ectopic vein formation.

## 5. Conclusions

The data presented in this work support our working hypothesis, proposing that the general co-repressors Gro and CtBP may be rate-limiting in specific developmental contexts exemplified for wing venation. Numerous transcriptional regulators recruit either one or both co-repressors Gro and CtBP, the activity of each being possibly impaired provided the limited availability of the co-repressors. Accordingly, the overexpression of any one of these regulators—for instance, Hairless—may induce dominant negative phenotypes by lowering co-repressor availability. These phenotypes, hence, do not necessarily reflect the function of the protein under investigation but are rather an impaired activity of other regulators inflicted by reduced co-repressor levels. We substantiated our working hypothesis for *H* in the context of wing vein development—*H* overexpression induced ectopic veinlet formation, which is normally masked by the manifold phenotypes—including wing phenotypes—arising from Notch inhibition, but uncovered by H* variants defective for Su(H) binding. As these H* variants cannot repress Notch signaling activity, the venation phenotype most likely reflects interference with other signaling pathways that result from depletion of co-repressors Gro and CtBP.

## Supplementary Materials

The following are available online at https://www.mdpi.com/2073-4425/11/10/1141/s1, Figure S1: Expression of H* mutant proteins, Figure S2: Phenotypic variance of wings from females derived from a cross with *omb*-Gal4, Figure S3: Phenotypic variance of wings from females derived from a cross with QE-Gal4.
