# Supplementary material for: Limited Availability of General Co-Repressors Uncovered in an Overexpression Context during Wing Venation in Drosophila melanogaster
Source: Genes (Basel). 2020 Sep 28;11(10):1141. doi: 10.3390/genes11101141 (PMC7601384; doi:10.3390/genes11101141)
Supplement: Supplementary file 1 [file genes-11-01141-s001.pdf]

Supplementary Material to

Limited availability of general co-repressors uncovered in an overexpression context during wing venation in *Drosophila melanogaster*

Anja C. Nagel [1,*], Dieter Maier [1], Janika Scharpf [1] , Manuela Ketelhut [1] and Anette Preiss [1]

[1] University of Hohenheim, Dept. of General Genetics 190g, Garbenstr. 30, 70599 Stuttgart, Germany
* Correspondence: anja.nagel@uni-hohenheim.de

The supplementary material contains three supplementary Figures S1-S3, including the methods applied and the relevant references.

Figure S1: Expression of H* mutant proteins

Figure S2: Phenotypic variance of wings from females derived from a cross with *omb*-Gal4

Figure S3: Phenotypic variance of wings from females derived from a cross with QE-Gal4.

References:

1. Kim, J.; Sebring, A.; Esch, J.J.; Kraus, M.E.; Vorwerk, K.; Magee, J.; Carroll, S.B. Integration of positional signals and regulation of wing formation and identity by *Drosophila vestigial* gene. *Nature* **1996**, *382*, 133-138.
2. Praxenthaler, H.; Nagel, A.C.; Schulz, A.; Zimmermann, M.; Meier, M.; Schmid, H.; Preiss, A.; Maier, D. Hairless-binding deficient Suppressor of Hairless alleles reveal Su(H) protein levels are dependent on complex formation with Hairless. *PLoS Genet.* **2017**, *13(5),* e1006774.
3. Zimmermann, M.; Kugler, S.J.; Schulz, A.; Nagel, A.C. Loss of *putzig* activity results in apoptosis during wing imaginal development in *Drosophila*. *PLoS One* **2015**, 10(4), e0124652.
4. Maier, D.; Praxenthaler, H.; Schulz, A.; Preiss, A. Gain of function notch phenotypes associated with ectopic expression of the Su(H) C-terminal domain illustrate separability of Notch and hairless-mediated activities. *PLoS One* **2013**, *8(11),* e81578.
5. developed by G. Rubin; obtained from the Developmental Studies Hybridoma Bank developed under the auspices of the NICHD and maintained by the University of Iowa, Dept. of Biology, Iowa City, IA 52242.
6. Smylla, T.K.; Meier, M.; Preiss, A.; Maier, D. The Notch repressor complex in *Drosophila*: in vivo analysis of *Hairless* mutants using overexpression experiments. *Dev Genes Evol.* **2019**, *229*, 13-24.

Figure S1: Expression of H* mutant proteins

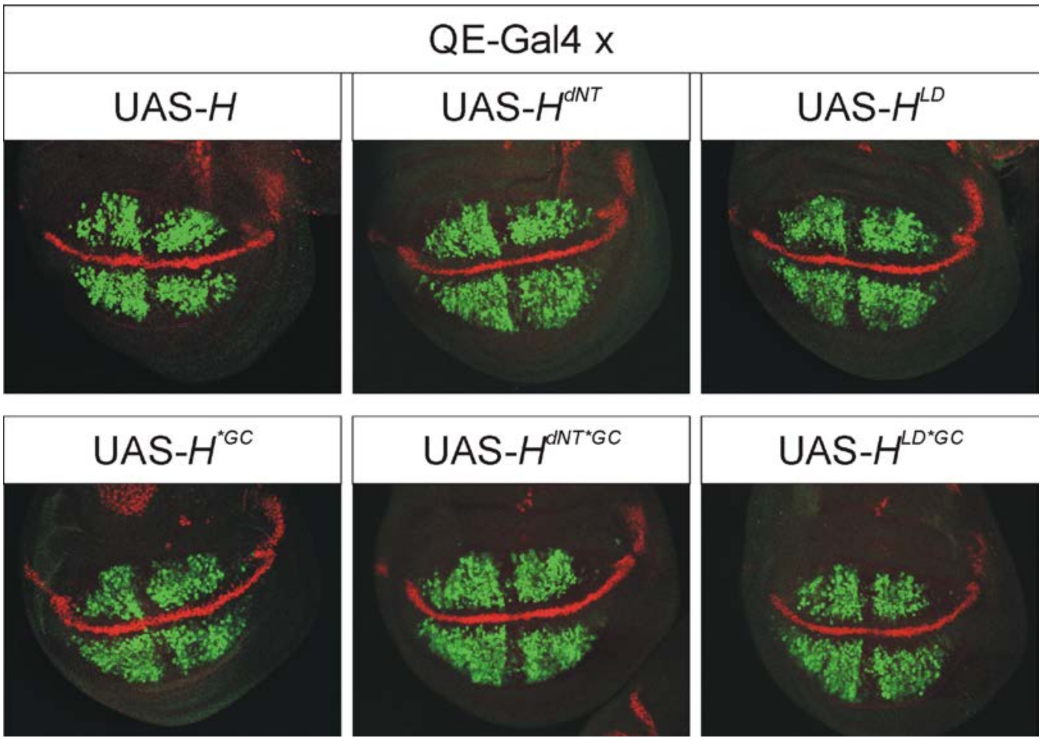

Expression of *H** UAS-lines in wing imaginal discs from third instar larvae derived from a cross of QE-Gal4 with the given UAS-line: UAS-*H*, UAS-H^{dNT}, UAS-H^{LD}, UAS-H^{*GC}, UAS-H^{dNT*GC}, UAS-H^{LD*GC}. Hairless expression is shown in green. The QE-Gal4 line drives expression in a quadrant pattern within the presumptive wing blade sparing antero-posterior and dorso-ventral boundaries [1]. To orient the reader, Cut protein expressed along the dorso-ventral boundary, is shown in red. Dorsal points up, and anterior to the right.

Immuno-cytochemistry on third instar imaginal discs was performed as described earlier [2,3]. We used guinea pig anti-Hairless A (1:500) [4] and mouse anti-Cut (1:25) [5] as primaries, and donkey secondary antibodies with minimal cross-reactivity, coupled to FITC and Cy3, respectively (Jackson Immuno-Research/Dianova, Hamburg, Germany). Vectashield (Vector labs, Eching, Germany) was used for mounting fluorescently labelled tissue. Pictures were taken with a Plan-NeoFluar 25x 1.3 Oil/Water objective on a Zeiss Axioskop coupled to a BioRad MRC1024 confocal microscope. The 488 nm and 568 nm laser lines were used for excitation of FITC and Cy3, respectively. *LaserSharp 2000TM* software (Carl Zeiss, Jena, Germany) allowed data acquisition, *PhotoPaint* and *CorelDraw* the assembly of pictures.

Figure S2: Phenotypic variance of wings from females derived from a cross with *omb*-Gal4.

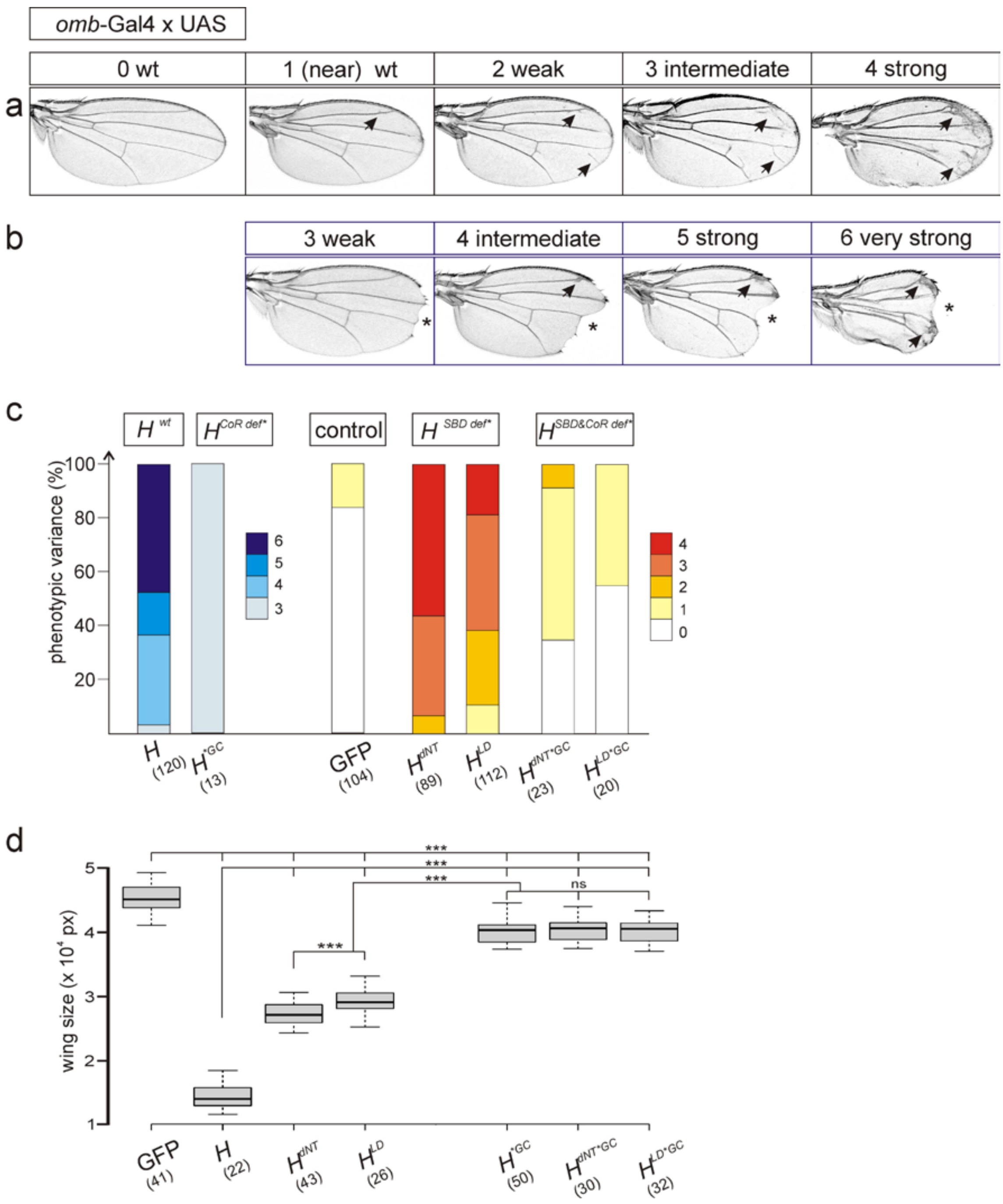

(a) Classification of phenotypes based on ectopic vein formation and vein thickening. Classes are: 0, wild typical wing. 1, nearly wild typical with occasional ectopic veinlets or a thickened vein. 2, weak phenotype with some ectopic veinlets usually along the L2 and L5 longitudinal veins. 3, intermediate phenotype with several ectopic veinlets. 4, strong phenotype characterized by a distal vein plexus. arrows point to thickened veins. Wing size was not taken into account and was measured separately in (d). Genotypes: *omb*-Gal4 x (0) UAS-GFP 18°C; (1) UAS-H^{LD} 18°C; (2) UAS-H^{LD} 18°C; (3) UAS-H^{dNT} 20°C; (4) UAS-H^{LD} 18°C.

(b) Classification of phenotypes based on wing incisions (asterisk) and size, as well as vein thickening (arrows). Classes are: 3, weak phenotype with several small notches. 4, intermediate phenotype with several, deeper notches and a thickening of longitudinal veins L2 and L3. 5, strong phenotype; wings are clearly smaller and have deep incisions and thickened veins. 6, very strong phenotype; wings are

less than half the normal size, have deep incisions and prominently thickened veins. Genotypes: *omb*-Gal4 x (3) UAS-H^{GC} 25°C; (4) UAS-*H* 18°C; (5) UAS-*H* 20°C; (6) UAS-*H* 25°C.

(c) Phenotypes were classified according to (a) and (b); the UAS-line analyzed is given below. Number of specimen is given in parentheses.

(d) Size of wings from females derived from a cross with *omb*-Gal4; the UAS-lines used are given below; number of data points is shown in parentheses. Wing size was measured using the freehand or ellipse tool of *Image J* as described earlier [6] and is given in pixel (px). The box plot was generated using the online application tool http://shiny.chemgrid.org/boxplotr/. Center lines show the medians; box limits indicate the 25th and 75th percentiles as determined by R software; whiskers extend 1.5 times the interquartile range from the 25th and 75th percentiles, outliers are represented by dots. Statistical analysis was conducted by ANOVA using a two-tailed Tukey-Kramer test for multiple comparisons. *** $p<0.001$ highly significant; ns, not significant $p>0.05$. Note tiny wings resulting from *H* overexpression. Wing size increased significantly when Su(H) binding deficient constructs were induced, and even more in the absence of co-repressor binding as well. The latter wings, however, are significantly smaller than the wild type control.

Figure S3: Phenotypic variance of wings from females derived from a cross with QE-Gal4.

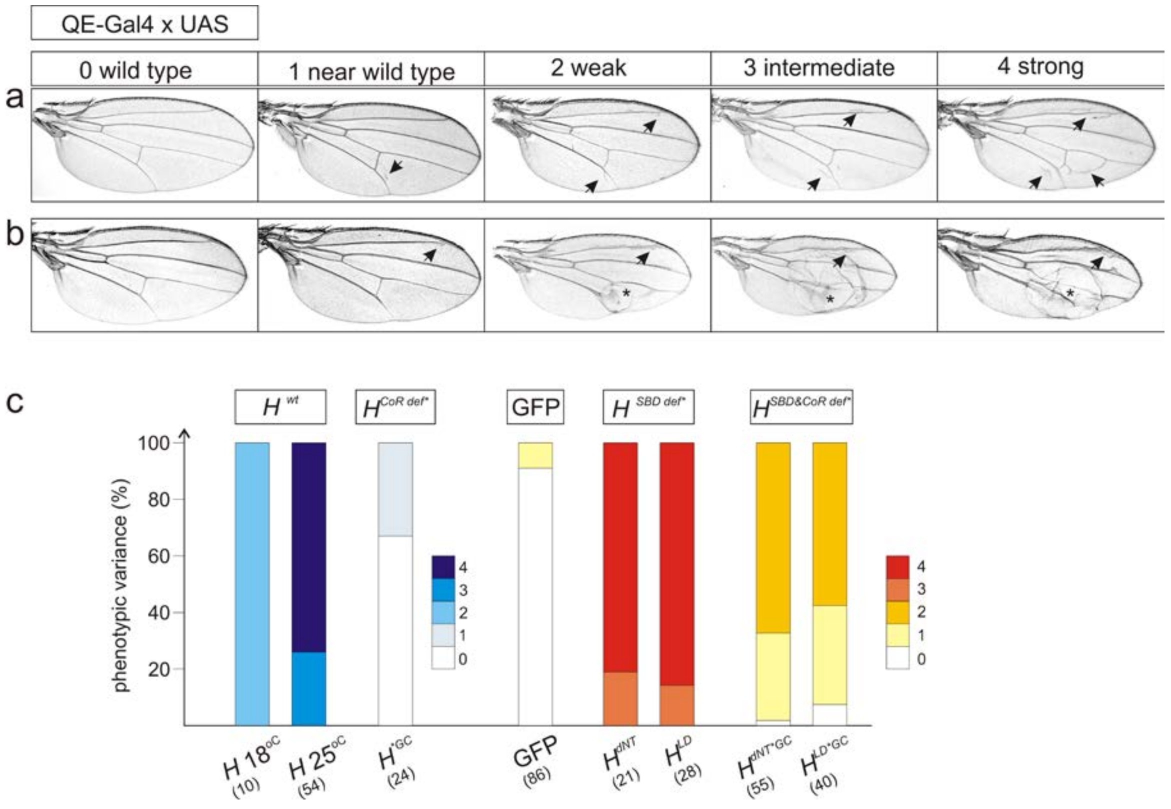

(a) Classification of phenotypes based on ectopic vein formation. Classes are: 0, wild typical wing. 1, nearly wild typical with remnants of ectopic veinlets. 2, weak phenotype with several remnants of ectopic veinlets usually along the L2 and L5 longitudinal veins. 3, intermediate phenotype with several ectopic veinlets a knotting along L2. 4, strong phenotype characterized by a distal vein plexus. Arrows point to ectopic veins. Genotypes: QE-Gal4 x (0) UAS-GFP 18°C; (1) UAS-GFP 25°C; (2) UAS-H^{dNT} 25°C; (3) UAS-H^{LD} 25°C; (4) UAS-H^{dNT} 25°C.

(b) Classification of phenotypes based on vein thickening (arrows) and size of blisters (asterisk). Classes are: 2, weak phenotype with knotted longitudinal L2 vein and a small blister at L5. 4, intermediate phenotype with knotted longitudinal L2 vein and a large blister at L5. 5, strong phenotype with several knotted longitudinal veins and a large blister at L5. Genotypes: QE-Gal4 x (0) UAS-H^{*GC} 25°C; (1) UAS-H^{*GC} 25°C; (2) UAS-*H* 18°C; (3) UAS-*H* 25°C; (4) UAS-*H* 25°C.

(c) Phenotypes were classified according to (a) and (b); the UAS-line analyzed is given below. Number of specimen is shown in parentheses.
